# Primary effusion lymphoma involving three body cavities

**DOI:** 10.4103/1742-6413.56361

**Published:** 2009-10-09

**Authors:** Fadi Brimo, Gizelle Popradi, René P Michel, Manon Auger

**Affiliations:** 1 Department of Pathology, McGill University and McGill University Health Center, Montreal, Quebec, Canada; 2 Division of Haematology, McGill University and McGill University Health Center, Montreal, Quebec, Canada

**Keywords:** Effusion, Epstein–Barr virus, human herpes virus-8, lymphoma

## Abstract

Primary effusion lymphoma (PEL) is a human herpes virus-8 (HHV8)-associated large-cell non-Hodgkin lymphoma localized in body cavities and presenting as pleural, peritoneal, or pericardial lymphomatous effusions. It typically affects immunocompromised patients and usually involves only one body site. We describe herein a case of PEL affecting three body cavity sites in an immunocompetent patient. A 69-year-old HIV-negative man presented with upper gastrointestinal bleeding and ascites. An examination of the fluid by cytology showed large atypical lymphocytes with abundant basophilic cytoplasm, either central or eccentric nuclei having irregular outlines, and multiple prominent nucleoli. The neoplastic cells showed positive staining for CD45, CD3, HHV8 latent nuclear antigen (LNA), and Epstein-Barr virus-encoded RNA. A diagnosis of PEL was rendered. Despite chemotherapy and valganciclovir, the disease progressed to involve the pleural and pericardial cavities and the patient died 5 months following the initial diagnosis. Although PEL is a B-cell lymphoma, it is usually of null phenotype by immunohistochemistry, and can rarely aberrantly express T-cell markers, as seen in the current case. The key to the diagnosis of PEL rests on identifying HHV8 in the neoplastic cells. Therefore, restricting the term of PEL only to those cases that are HHV8 positive is important in order to differentiate PEL from other lymphomas that can present as serous effusions and that carry, in general, a more favorable prognosis than PEL

## INTRODUCTION

In 2001, primary effusion lymphoma (PEL) was first introduced as an officially recognized and distinct entity in the World Health Organization (WHO) classification of neoplastic diseases of the hematopoietic and lymphoid tissues[1] PEL refers to a large-cell non-Hodgkin lymphoma localized in body cavities and presenting as pleural, peritoneal, or pericardial lymphomatous effusions. PEL is considered to be always associated with human herpes virus-8 (HHV8) infection; therefore, this term is restricted to those lymphomatous effusions that are associated with HHV8[1–4] It has been shown that the presence of the virus in the neoplastic cells can be demonstrated either by immunohistochemical methods using the HHV8 LNA-1 latent protein antibody, or by molecular techniques such as PCR amplification or Southern blot analysis[4–12] PEL typically involves only one body site, the most common being the pleural cavity; however, involvement of two body cavity sites has been reported in some series[1,2,4] Although the majority of PEL cases affect human immunodeficiency virus (HIV)-positive patients, some cases have been reported in cancerous and cirrhotic patients or following solid organ transplantation, presumably leading to an immunosuppressed state in them[13–17] Rarely, PEL develops in immunocompetent populations in which it affects older individuals living in geographical areas with a high prevalence of HHV8 infection[3,18].

We describe herein one case of PEL affecting three body cavities in an immunocompetent male.

## CASE REPORT

### Clinical summary

A 69-year-old HIV-negative man of Greek origin presented with new onset of abdominal distension, lower extremity edema, dyspnea, nonproductive cough, and upper gastrointestinal bleeding. Past medical history included schizophrenia, diabetes mellitus, hypertension, renal insufficiency, and atrial fibrillation. Clinical examination revealed ascites in the absence of palpable adenopathy or hepatosplenomegaly. Complete blood count, liver function tests, lactate dehydrogenase, and serum protein were within normal limits. Cytological examination of peritoneal fluid revealed an abnormal lymphoid population consistent with PEL.

Further investigations confirmed the HIV-negative status, established that the patient did not have hepatitis B or C, and demonstrated normal immunoglobulin levels and the absence of a serum monoclonal protein. Staging studies including CT scans of the abdomen and thorax were performed and showed a noncirrhotic liver and the absence of lymphadenopathy or organ involvement by lymphoma. The patient was started on anthracycline-based multiagent chemotherapy (CHOP; cyclophosphamide, doxorubicin, vincristine, and prednisone) plus continuous daily oral valganciclovir. He tolerated the chemotherapy and had a satisfactory clinical response following which he was able to return home on daily valganciclovir. However, shortly thereafter, he returned to the hospital with recurrent ascites and nonobstructive ileus. A 6-l paracentesis was performed on which cytology showed persistent PEL. At this time, a small, right-sided effusion was noted on the chest radiograph. Following a prolonged admission and convalescence, the patient's condition was felt to be stable and he was discharged with a tentative plan of a third cycle of CHOP. However, within weeks of discharge, the patient was readmitted with acute respiratory failure requiring noninvasive ventilation. Investigations revealed bilateral pleural effusions and a large pericardial effusion. Following a therapeutic thoracentesis and a mini-thoracotomy for drainage of the pericardial effusion, the patient was able to breathe without supplemental oxygen. Cytology from both the pericardial and pleural fluids was again consistent with PEL. Thus a diagnosis of PEL involving three body cavities (pericardial, pleural, and peritoneal) was made. After discussion of the poor prognosis and rapid progression of the patient's malignancy, a supportive care strategy was adopted. He died within 5 months of the initial diagnosis of PEL.

### Cytomorphological, immunocytochemical, and *in situ* hybridization findings

**Figure 1:**
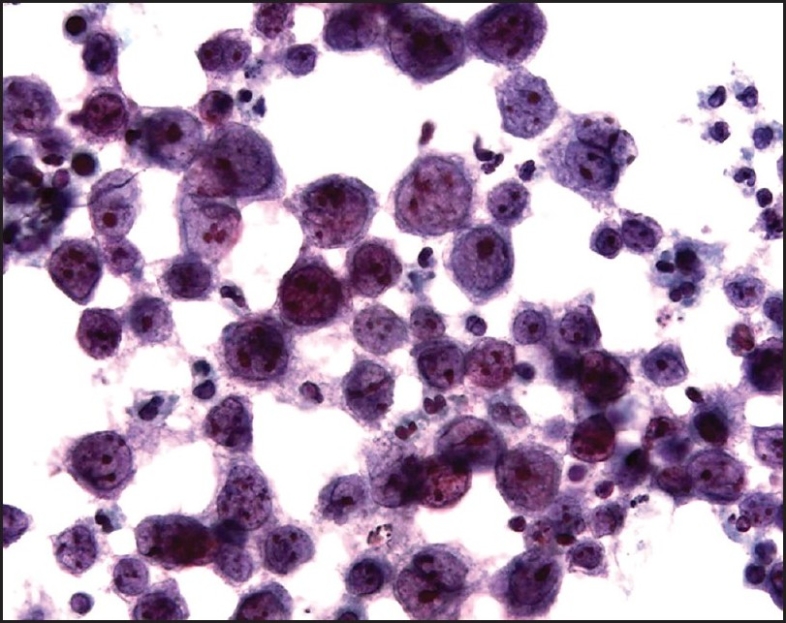
Cytology of the peritoneal fluid. Note the large cell size, the moderately abundant basophilic cytoplasm, the eccentric nuclear location in some cells, and the prominent nucleoli. Apoptotic bodies are also present (cytospin preparation, Papanicolaou stain, ×600)

**Figure 2:**
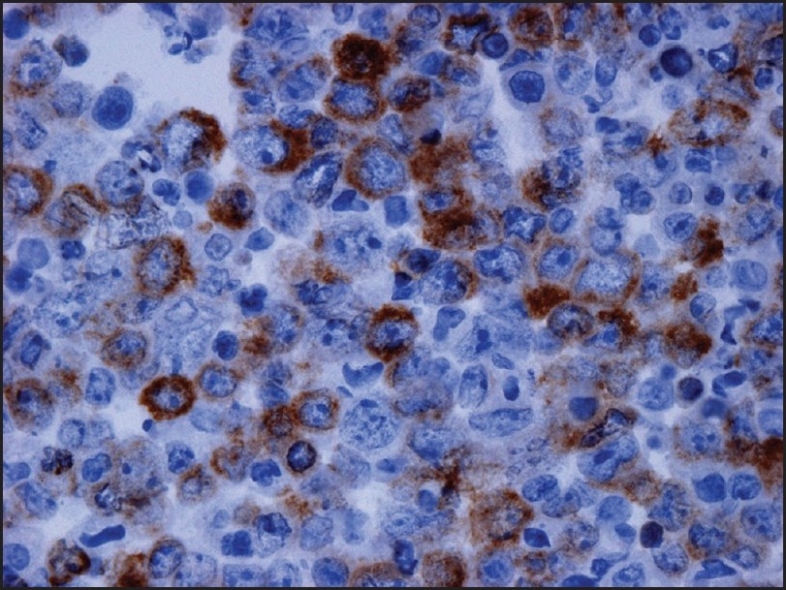
Immunocytochemistry performed on the cell block of the peritoneal fluid shows positive staining in the malignant cells for cytoplasmic CD3 (×400)

**Figure 3:**
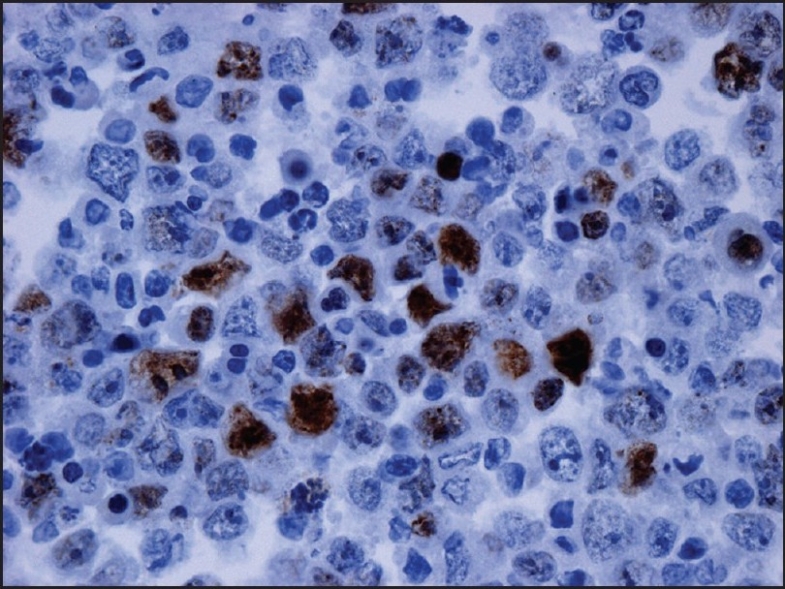
Immunostain for human herpes virus-8 performed on the cell block of the peritoneal fluid shows positivity in 25–30% of the malignant cells (×400)

Examination of the initial peritoneal fluid specimen revealed a hypercellular smear composed of large atypical lymphocytes with abundant basophilic cytoplasm. Although some cells displayed a central nuclear location, others contained eccentrically located nuclei with a plasmablastic appearance. The nuclei showed irregular outlines and contained coarse chromatin and multiple prominent nucleoli. Numerous apoptotic bodies and abundant nuclear debris were also noted [Figure 1]. Immunohistochemical studies showed positive staining of the neoplastic cells for CD45 and cytoplasmic staining for the T-cell marker CD3 in all specimens [Figure 2]; staining for CD2, CD4, CD5, CD7, and CD43 was negative. Immunostains for the B-cell markers CD20, CD79a, and PAX-5 were negative, as were ALK-1, bcl 2, bcl 6, CD10, CD138, and keratin AE1/3. Staining for Ki67 showed positivity in about 80% of the nuclei. Using an antibody directed against HHV8 latent nuclear antigen (LNA), there was positive staining in 25–30% of the neoplastic lymphocytes [Figure 3]. In addition, *in situ* hybridization for Epstein-Barr virus-encoded RNA (EBER) done on one of the cell blocks showed positivity in about 50–60% of the nuclei. Similar morphological and immunocytochemical findings were noted in the fluid specimens that were subsequently taken for cytology from the pleural and pericardial cavities. The percentage of HHV8-positive cells remained unchanged in the peritoneal fluid pre- and postchemotherapy (25–30% of cells staining), and it subsequently increased to 50% in the pleural fluid specimen that was taken 5 months following the initial diagnosis.

## DISCUSSION

PEL is a large-cell lymphoma with morphological features bridging immunoblastic and anaplastic large-cell lymphomas (ALCL).[3,4] Although it is generally of indeterminate phenotype immunohistochemically, it is considered to be a B-cell lymphoma by molecular studies.[1–4,9] It has been suggested that PEL is derived from postgerminal center B-cells approaching plasma cell differentiation, which explains its frequent expression of activation and plasma cell markers such as CD30, CD38, CD138, and EMA.[19–21] Rarely, PEL aberrantly expresses T-cell markers or demonstrates T-cell receptor gene rearrangement.[4,22,23] Of note, of the five cases of PEL identified in our institution to date, three (including the current case) were positive for T-cell markers by immunohistochemistry.[4]

Independent of the immunoprofile that cells from PEL display in individual cases, the key to diagnosis rests on identifying HHV8 in the neoplastic cells, using either immunohistochemical or molecular techniques.[1–4] We believe that the term PEL should be restricted to those HHV8-positive lymphomatous effusions only, as originally proposed in the 2001 edition of the WHO.[1] Recently, different and somewhat confusing terminologies have emerged in the literature to describe lymphomatous effusions in which HHV8 was absent, the most commonly used terminology being “HHV8-unrelated PEL-like lymphoma.”[24–26] Such cases usually affect HIV-negative patients and are associated with hepatitis C virus (HCV) and Epstein-Barr virus (EBV) in up to 42% and 19% of the time, respectively.[24–27] Due to the fact that the most commonly involved site in these cases is the peritoneum, it has been suggested that HCV may play a pivotal pathogenic role in these cases by causing persistent antigenic stimulation leading to the development of an intraperitoneal clonal B-cell proliferation.[27] In addition, “HHV8-unrelated PEL-like lymphoma” cases commonly express B-cell markers (90% of cases) in contrast to PEL.[3,26] These data combined with the reported better prognosis of ‘HHV8-unrelated PEL-like lymphoma” in comparison to PEL, strongly suggest that these two entities are clearly distinct.[26] Moreover, it has been shown that some types of lymphomas such as diffuse large B-cell lymphoma, ALCL, and Burkitt lymphoma can present rarely as serous effusions.[28–31] Therefore, it is likely that the so-called HHV8-unrelated PEL-like lymphoma entity represents a heterogeneous group of lymphomas affecting serous cavities rather than being a distinct and well-defined entity like PEL.

Another caveat worthy of mention is that PEL is now considered part of the spectrum of HHV8-related lymphomas. These are divided into PEL when the site of involvement is the serous cavities, and “solid PEL” or “extracavitary PEL” which represents an HHV8-associated lymphoma presenting primarily as a solid mass without serous effusions, or preceding the development of an effusion lymphoma.[21,32,33]

To date, there is no standard treatment recommended for PEL, mainly due to its rarity. Routinely, CHOP (cyclophosphamide, doxorubicin, vincristine, and prednisolone)-like regimens are used, but the clinical outlook is generally extremely unfavorable, with a median survival of less than 6 months.[2] HAART (highly active antiretroviral therapy) has been rarely used alone in the treatment of HIV-associated PEL, and some studies have reported long-term survival up to 31 months following the diagnosis.[34,35] Other therapeutic approaches include antiviral medications with activity against EBV such as ganciclovir or intracavitary cidofovir, used alone or in combination with chemotherapy. Using these agents, durable remissions have been reported in some series.[36–39] In the present case, valganciclovir, an oral prodrug of gancyclovir that is easy to administer, was combined with chemotherapy based on recent data suggesting that it prevents HHV8 replication *in vivo*.[40] Despite this, the patient's disease progressed and spread from an apparent primary peritoneal site to three body cavities, i.e., pleural, peritoneal, and pericardial. Of note, the percentage of cells staining with the HHV8 antibody (25–30%) in the peritoneal fluid did not change despite chemotherapy, and indeed rose to 50% in the pleural fluid 5 months following the initial diagnosis. Whether the extent of staining of the neoplastic cells with the HHV8 antibody correlates with the response to treatment has not been studied and remains unknown.

## CONCLUSION

Primary effusion lymphoma (PEL) is an HHV8-related lymphoma that can display variable immunocytochemical profiles. Therefore, restricting the PEL terminology to those cases that are HHV8 positive is important to differentiate PEL from other lymphomas that can present as serous effusions and carry in general a more favorable prognosis.

## COMPETING INTEREST STATEMENT BY ALL AUTHORS

No competing interest to declare by any of the authors.

## AUTHORSHIP STATEMENT MADE BY ALL AUTHORS

Each author acknowledges that this final version was read and approved. According to the International Committee of Medical Journal Editors (ICMJE http://www.icmje.org) “author” is generally considered to be someone who has made substantive intellectual contributions to a published study. Authorship credit should be based on (1) substantial contributions to conception and design, acquisition of data, or analysis and interpretation of data; (2) drafting the article or revising it critically for important intellectual content; and (3) final approval of the version to be published. Authors should meet conditions (1), (2), and (3). Other contributors, who do not meet these criteria for authorship, are listed in an “acknowledgments” section. All authors of this article declare that we qualify for authorship as defined by ICMJE http://www.icmje.org/#author. Each author has participated sufficiently in the work and takes public responsibility for appropriate portions of the content of this article.

## ETHICS STATEMENT BY ALL AUTHORS

As this is a case report without patient identifiers, approval from *Institutional Review Board* (IRB) is not required at our institution.
